# Association of rare haplotypes on *ULK4* and *MAP4* genes with hypertension

**DOI:** 10.1186/s12919-016-0057-2

**Published:** 2016-11-15

**Authors:** Ananda S. Datta, Yuan Zhang, Lei Zhang, Swati Biswas

**Affiliations:** Department of Mathematical Sciences, University of Texas at Dallas, Richardson, TX USA

## Abstract

Several variants have been implicated earlier on *ULK4* and *MAP4* genes on chromosome 3 to be associated with hypertension. As a natural follow-up step, we explore association of haplotypes in those genes. We consider the Genetic Analysis Workshop 19 real data on unrelated individuals and analyze haplotype blocks of 5 single-nucleotide polymorphisms through a sliding window approach. We apply 4 haplotype association methods—haplo.score, haplo.glm, hapassoc, and logistic Bayesian LASSO (LBL)—and for comparison, sequence kernel association test (SKAT) and its variants. We find several rare haplotype blocks to be associated. To get an idea about the false-positive proportions, we also analyzed the data after permuting the case-control status of individuals. We found that LBL, unlike the other methods, maintains low false-positive rates in presence of rare haplotypes. Thus, we conclude that the haplotypes found to be associated by LBL are more likely to be true positive. SKAT and its variants did not find significance on either gene.

## Background

Past studies have implicated several variants on chromosome 3, in particular, on genes *ULK4* and *MAP4*, as being associated with blood pressure and hypertension [1–9]. A typical follow-up step is to zoom into these regions through haplotype association analyses. Haplotype-based methods can be more powerful than single single-nucleotide polymorphism (SNP) methods especially when the causal variants are not genotyped or multiple variants act in *cis* [10–12]. In some situations, they also have increased power over the recently developed popular “collapsing” methods for detecting rare variant associations [13–15]. The availability of Genetic Analysis Workshop (GAW) 19 exome sequencing data on hypertension provides such an opportunity [16]. However, a majority of SNPs in the GAW19 data set are rare; for example, less than 3 % of variants on chromosome 3 have a minor allele frequency (MAF) of 0.01 or more, so when rare SNPs are combined to form haplotype blocks, the haplotypes will be even rarer. Thus, it is important to use a haplotype association method that can handle rare haplotypes.

Logistic Bayesian LASSO (least absolute shrinkage and selection operator) (LBL) has been proposed for detecting rare haplotype association and has shown promising results in both real and simulated data sets [17–19]. By regularizing the regression coefficients through their prior distributions, LBL weeds out unassociated (especially common) haplotypes, allowing the associated rare haplotypes to be more easily detected. Extensive simulation studies, including those on GAW18 data [19], have shown that LBL has good power to detect associated haplotypes (rare as well as common) while maintaining low type I error rates. Thus, we choose to use this method for studying haplotype association in this article. Additionally, we also use 3 standard and widely used haplotype association methods—haplo.score [20] and haplo.glm [21] implemented in R package haplo.stats, and hapassoc [22], another R package.

## Methods

### Statistical methods for haplotype association

The three standard approaches considered here—haplo.score, haplo.glm, and hapassoc—are based on the generalized linear model (GLM). In haplo.score, a global test of association as well as individual haplotype-specific tests are carried out using a score function. It estimates haplotype frequencies independently of trait or covariates under the null hypothesis of no association. Haplo.score does not estimate the magnitude of individual haplotype effects. Haplo.glm is an extension of haplo.score for testing haplotype–environment interactions (it can fit a main-effects-only model also). Unlike haplo.score, it iteratively estimates haplotype frequencies conditional on all observed data and current estimates of regression parameters. It uses Wald tests for testing a global haplotype–environment interaction effect and individual haplotype-specific effects. Also, it estimates the magnitude of individual haplotype effects [21]. Hapassoc was proposed as an extension of haplo.glm to accommodate missing genotype data at individual SNPs (although haplo.glm can now accommodate missing genotypes) and uses an improved approximation to standard error estimation [22]. All of these methods can handle binary as well as continuous response.

As the above three approaches are not specifically designed for rare haplotypes, they may or may not perform well in presence of rare haplotypes. Indeed, in previous studies [17–19], hapassoc has shown high non-convergence rates when rare haplotypes are modeled individually rather than pooled together, which is a typical approach for handling rare haplotypes but one that doesn’t allow study of individual rare haplotypes. Thus, we also apply LBL, which is described in details in Biswas and Lin [17] and Biswas et al [18], and briefly here.

LBL is based on a retrospective likelihood; that is, it models the probability of haplotypes given disease status. The unobserved (phased) haplotypes of subjects are treated as missing data and frequencies of haplotype pair for each person are modeled using haplotype frequencies (treated as unknown parameters) and allowing for Hardy-Weinberg disequilibrium. The odds of disease are expressed as a logistic regression model, whose coefficients are regularized through a double-exponential prior centered at zero and a variance parameter, which is further assigned a hyper prior. This regularization corresponds to the Bayesian LASSO. Markov chain Monte Carlo methods are used for estimating the posterior distributions of all parameters, which include regression coefficients and haplotype frequencies. Testing for association for each main and interaction effect is carried out by calculating the Bayes factor (BF). A BF exceeding 2 is considered significant evidence of association. The posterior mean and confidence intervals of parameters can be obtained, if desired. LBL is available as an R package at http://www.utdallas.edu/~swati.biswas/. Currently, LBL can only handle binary (case-control) responses.

### Selection of regions and data for analysis

We consider 2 genes—*ULK4* and *MAP4*. We exclude SNPs with more than 25 % of genotypes missing and include SNPs with a MAF of at least 0.001. We use sliding and overlapping windows made up of 5 SNPs to create haplotype blocks (eg, SNPs 1 to 5, 2 to 6, and so on) to cover the whole gene.

For selection of SNPs and calculation of MAF, we used genotypes listed under NALTT (the number of alternate alleles thresholded), coded as 0/1/2; these are high-quality genotypes. An alternate allele is usually the minor allele (but not always); for simplicity, we coded the major allele as 0 and minor allele as 1. For phenotype, we defined a binary hypertension trait as follows. If a person has systolic blood pressure (SBP) greater than 140 or diastolic blood pressure (DBP) greater than 90 or is taking antihypertensive medication, we labeled that person to be affected by hypertension (case). Otherwise, the individual is labeled as unaffected (control). Also, a person with SBP and DBP values below thresholds and whose medication field is missing is treated as a control.

We apply all four methods on the above described haplotype blocks without using any covariates. For LBL, we use a threshold of BF greater than 2, whereas for other methods we use a *p* value of less than 0.05 to declare significance. We analyze blocks in each gene twice—using the provided phenotypes and after randomly permuting the phenotype status among all subjects. The latter destroys association, if there is any, and so allows us to gauge the false-positive rates. Finally, we also analyzed using LBL after including in the model the covariate age (dichotomized at 55) and its interaction with haplotypes.

To allow for rare haplotypes to be analyzed individually, and not be pooled together, we set the pooling tolerance of hapassoc to zero, where pooling tolerance is a value (user-defined) of haplotype frequency below which the corresponding haplotypes are pooled into a single category called pooled in the design matrix for the risk model. In the hapassoc package, there is a pre-processing function called pre.hapassoc, which returns a list of compatible haplotypes for each person’s genotypes and frequencies of all haplotypes in the population. These are provided as input to hapassoc and LBL. In LBL, the estimated frequencies of haplotypes are used as starting values of frequency parameters. Haplo.glm does not allow pooling tolerance to go below 0.001. For a fair comparison of haplo.glm and hapassoc, we also ran hapassoc with pooling tolerance of 0.001. Haplo.score does not pool any haplotypes. Finally, for comparison purpose, we also analyzed each gene (all SNPs within a gene together) using popular collapsing approaches of sequence kernel association test (SKAT), SKAT-Optimal (SKAT-O), and SKAT-Combined (SKAT-C) [23–25].

## Results

The total numbers of cases and controls are 456 and 1395, respectively (*n* = 1851) after excluding subjects with missing disease status. We report the results for *ULK4* and *MAP4* genes separately.

### *ULK4* gene

There are 70 SNPs. and so, with a sliding window of 5 SNPs, we analyzed a total of 66 haplotype blocks. A significant haplotype was found by at least one of the methods in 36 blocks. Using LBL, we found evidence for association in 18 blocks, as shown in Table 1. These blocks are in the regions 412910181 (SNP 3) to 41759191 (SNP 22) bp and 419425423 (SNP 39) to 41949348 (SNP 48) bp. In particular, the blocks 40 to 44 and 42 to 46 have haplotypes with extremely strong evidence of association with BF greater than 100. However, in these and some other blocks in Table 1, haplo.glm or haplo.score results were not significant. In Table 2, we report the haplotypes found to be significant by either of these two methods but not by LBL. Hapassoc with pooling tolerance of zero converged in only six blocks, and was significant in three blocks starting with SNPs 6, 7, and 9. With pooling tolerance of 0.001, it converged in 15 more blocks; in that case, its results were similar to that of haplo.glm, which converged in all blocks. When LBL was analyzed by including age and its interaction with haplotypes, some of the haplotypes found significant earlier with main effects only model were still significant (but not all of them). Additionally, we found significant interactions of age with haplotypes in the region covered by SNPs 60 to 69 (41960004 to 41996136). Interestingly, these interactions are protective (odds ratio [OR] < 1) and their main effects are not significant (same holds in the main-effects-only model). The main effect of age was also significant. SKAT and its variants did not show significance in this gene. The *p* values for SKAT, SKAT-C and SKAT-O are 0.170, 0.239, and 0.258, respectively.

**Table 1:** Significant haplotypes on *ULK4* gene by LBL

SNP# in haplotype block	Location	Hap name	Hap freq	LBL (OR)	LBL (BF)	Haplo GLM (*p* value)	Haplo score (*p* value)	Haplo score overall test (*p* value)
3–7	41291081–41497081	10101	0.0014	3.823	3.232*	0.023*	0.004*	0.204
4–8	41439551–41497115	01010	0.0012	6.064	5.627*	0.000*	0.001*	0.016*
5–9	41439790–41504594	10101	0.0012	5.920	6.796*	0.000*	0.001*	0.012*
6–10	41439797–41504679	01010	0.0014	3.477	2.909*	0.000*	0.004*	0.008*
7–11	41497081–41607541	10100	0.0013	3.490	2.511*	0.000*	0.004*	0.009*
8–12	41497115–41657184	01000	0.0014	3.511	3.061*	0.000*	0.004*	0.005*
9–13	41504594–41722969	10000	0.0014	3.314	2.511*	0.024*	0.004*	0.005*
11–15	41607541–41723054	00010	0.0019	0.218	2.020*	0.000*	0.130	0.085
15–19	41723054–41756933	00010	0.0125	0.384	4.736*	0.032*	0.019*	0.129
16–20	41723090–41756965	00101	0.0122	0.382	4.599*	0.029*	0.022*	0.125
17–21	41723151–41756986	01011	0.0121	0.385	4.254*	0.032*	0.022*	0.191
18–22	41723280–41759191	10111	0.0118	0.358	7.985*	0.019*	0.015*	0.128
39–43	41925423–41939990	00001	0.0055	2.512	3.396*	0.012*	0.004*	0.219
40–44	41937000–41939992	10000	0.0935	2.101	3.930*	0.282	0.132	0.180
		00010	0.0050	6.598	>100*	0.048*	0.002*	
		00100	0.0466	4.586	>100*	0.234	0.121	
		00101	0.0769	0.546	2.456*	0.668	0.314	
41–45	41938500–41942199	00011	0.0458	2.623	6.006*	0.185	0.895	0.357
42–46	41938522–41942348	01000	0.0062	2.166	2.031*	0.235	0.024*	0.157
		00110	0.0443	3.462	>100*	0.325	0.743	
43–47	41939990–41949301	01101	0.0413	2.285	4.025*	NA	0.887	0.428
44–48	41939992–41949348	11010	0.0418	2.201	3.677*	0.525	0.807	1.000

Major allele is coded as zero. SNP# corresponds to the order of SNP in the gene among SNPs with MAF ≥0.001 and no more than 25 % missing genotypes

*Hap* haplotype, *Hap freq* haplotype frequency (obtained from hapassoc); NA, haplo.glm did not run for this region and gave an error

*Significant BF or *p* value

**Table 2:** Significant haplotypes on *ULK4* gene by haplo.glm or haplo.score (in addition to those indicated in Table 1)

SNP# in haplotype block	Location	Hap name	Hap freq	LBL (OR)	LBL (BF)	Haplo GLM (*p* value)	Haplo score (*p* value)	Haplo score overall test (*p* value)
**8–12**	41497115–41657184	00001	0.0014	2.471	1.430	0.040*	0.010*	0.005*
**9–13**	41504594–41722969	00010	0.0016	2.022	1.123	0.035*	0.016*	0.005*
10–14	41504679–41722976	00100	0.0016	2.155	1.050	0.036*	0.016*	0.064
		00001	0.0019	0.218	1.743	0.000*	0.130	0.064
11–15	41607541–41723054	01000	0.0016	2.118	1.394	0.037*	0.016*	0.085
		00001	0.0011	0.305	1.151	0.000*	0.252	0.085
12–16	41657184–41723090	10000	0.0016	2.137	1.281	0.036*	0.016*	0.041*
		00010	0.0011	0.307	1.278	0.000*	0.252	0.041*
		00100	0.0019	0.226	1.773	0.000*	0.130	0.041*
26–30	41796016–41841618	00001	0.0033	2.036	1.340	0.047*	0.040*	0.331
		10000	0.0016	0.286	1.355	0.000*	0.161	0.331
27–31	41796025–41841716	00010	0.0032	1.945	1.051	0.045*	0.040*	0.565
28–32	41831203–41841811	00100	0.0032	1.900	0.989	0.047*	0.040*	0.357
		00001	0.0018	0.248	1.595	0.000*	0.132	0.357
30–34	41841618–41861013	10000	0.0030	1.877	1.036	0.039*	0.046*	0.649
		01001	0.0016	0.565	0.737	0.000*	0.487	0.649
**39–43**	41925423–41939990	01010	0.0012	0.555	0.809	0.000*	0.543	0.219
**40–44**	41937000–41939992	10010	0.0016	0.821	0.798	0.000*	0.495	0.253
		10100	0.0012	0.606	0.791	0.000*	0.549	0.253
**44–48**	41939992–41949348	01001	0.0004	2.228	1.270	NA	0.000*	1.000
		10001	0.0010	1.813	0.915	0.000*	0.104	1.000
47–51	41949301–41952774	00001	0.0048	1.881	1.407	0.046*	0.043*	0.000*
48–52	41949348–41952781	00010	0.0048	1.842	1.252	0.049*	0.040*	0.011*
49–53	41949359–41952838	00100	0.0048	1.900	1.293	0.049*	0.040*	0.161
50–54	41949479–41952852	01000	0.0047	2.000	1.449	0.032*	0.037*	0.299
51–55	41952774–41952898	10000	0.0045	2.163	1.838	0.023*	0.023*	0.218

Major allele is coded as 0. SNP# corresponds to the order of SNP in the gene among SNPs with MAF ≥0.001 and no more than 25 % missing genotypes. The blocks shown in bold in the first column are reported in Table 1 also but for a different haplotype

*Hap* haplotype, *Hap freq* haplotype frequency (obtained from hapassoc); NA, this haplotype was not returned by haplo.glm as its frequency is below pooling tolerance of 0.001

*Significant *p* value

### *MAP4* gene

There are 18 SNPs and so there is a total of 14 of the 5-SNP haplotype blocks. A significant haplotype was found by at least 1 of the methods in 10 blocks. With LBL, we found association in 1 block only (Table 3) in the region covered by SNPs 11 to 15 (47956424 to 47969734 bp). Table 4 shows that haplo.glm found association in nine additional blocks in the regions formed by SNPs 2 to 13 (47910743 to 47958037 bp). However, haplo.score only found one of these nine blocks to be significant (the block starting with SNP 8). Hapassoc with pooling tolerance of zero converged in six blocks, and was significant in three blocks starting with SNPs 7, 8, and 10. With a pooling tolerance of 0.001, it converged in one more block and the results were very similar to those of haplo.glm. When we include age and its interaction in LBL, age was significant, but none of the interaction terms were significant. We did not find any significant association using SKAT, SKAT-O, and SKAT-C whose *p* values are 0.717, 0.250, and 0.802, respectively.

**Table 3:** Significant haplotypes on *MAP4* gene by LBL

SNP# in haplotype block	Location	Hap name	Hap freq	LBL (OR)	LBL (BF)	Haplo GLM (*p* value)	Haplo score (*p* value)	Haplo score overall test (*p* value)
11–15	47956424–47969734	10000	0.0041	2.467	3.190*	0.011*	0.010*	0.089

Major allele is coded as zero. SNP# corresponds to the order of SNP in the gene among SNPs with MAF ≥0.001 and no more than 25 % missing genotypes

*Hap* haplotype, *Hap freq*, haplotype frequency (obtained from hapassoc)

*Significant BF or *p* value

**Table 4:** Significant haplotypes on *MAP4* gene by haplo.glm or haplo.score (in addition to those indicated in Table 2)

SNP# in haplotype block	Location	Hap name	Hap freq	LBL (OR)	LBL (BF)	Haplo GLM (*p* value)	Haplo score (*p* value)	Haplo score overall test (*p* value)
2–6	47910743–47917263	00001	0.0011	0.358	1.095	0.000*	0.252	0.643
3–7	47912703–47950634	00010	0.0011	0.369	1.090	0.000*	0.252	0.412
4–8	47913380 –47951234	00100	0.0011	0.358	1.102	0.000*	0.252	0.457
5–9	47913498–47951238	01000	0.0011	0.345	1.211	0.000*	0.252	0.411
6–10	47917263–47951299	10000	0.0011	0.345	1.113	0.000*	0.252	0.257
7–11	47950634–47956424	00001	0.3347	1.155	0.272	0.044*	0.065	0.097
8–12	47951234–47957996	00010	0.3131	1.175	0.368	0.029*	0.034*	0.203
10–14	47951299–47963395	01010	0.2828	1.156	0.250	0.048*	0.086	0.177
**11–15**	47956424–47969734	10100	0.2860	1.172	0.324	0.040*	0.058	0.089
13–17	47958037–48040283	10000	0.2782	1.169	0.312	0.047*	0.051	0.614

Major allele is coded as zero. SNP# corresponds to the order of SNP in the gene among SNPs with MAF ≥0.001 and no more than 25 % missing genotypes. The block shown in bold in the first column is reported in Table 3 also but for a different haplotype

*Hap* haplotype, *Hap freq* haplotype frequency (obtained from hapassoc)

*Significant *p* value

### False-positive rates

As described in the Methods section above, a null scenario was created by permuting the case-control status of subjects. In the following false-positive rates, the denominator is the total number of haplotypes in all haplotype blocks of a gene reported by each method and the numerator is the number of haplotypes found to be significant among them. Furthermore, for each method, we report 2 rates in the order of *ULK4* and *MAP4* genes. LBL: 10/510 = 2 % and 0/81 = 0 %; haplo.glm: 26/358 = 7.26 % and 3/72 = 4.16 %; haplo.score (individual haplotype test): 28/420 = 6.67 % and 0/74 = 0 %; haplo.score (overall test): 8/66 = 12.12 % and 0/14 = 0 %. Note that different methods report different numbers of haplotypes in a block. Haplo.glm has smallest denominator as it pools haplotypes with frequencies below 0.001 into 1 pooled haplotype. We don’t report this rate for hapassoc as it does not converge in most cases. Also note that strictly speaking, these rates are not correct estimates of type I error rates as the tests for different haplotypes on same/different blocks are not independent replications of a single test. Nonetheless, these do give us an idea about the true false-positive rates, at least qualitatively.

## Discussion

We have found significant haplotype association on *ULK4* and *MAP4* genes. Most of these are rare haplotypes with frequencies less than 2 %. Because of presence of rare haplotypes, hapassoc did not converge most of the time. Haplo.glm, with its minimum pooling tolerance of 0.001, gave the maximum number of significant haplotypes, followed by haplo.score. However, we found that these standard methods tend to give inflated false-positive rates in the presence of rare haplotypes. We have found this trend in our own simulations also using different data sets (not presented here). Thus, caution is warranted in treating the associated haplotypes shown by these methods as true positive.

On the other hand, we found that LBL maintains low type I error rates in presence of rare haplotypes, and this was also shown in previous studies including GAW18 simulated data [17–19]. So, the significant results from LBL are more likely to be true positive, especially those with a large BF. We also created haplotype blocks using Haploview [26] based on the CEU (Northern Europeans from Utah) population from the International Haplotype Map Project (HapMap) Project Phase 3. Some of the regions found to be significant by LBL fall in those blocks, in particular, SNPs 4 to 6, 7 to 10, and 39 to 48 on the *ULK4* gene, and the significant haplotype on *MAP4* gene. On incorporating age and its interaction effects, LBL found some interaction effects to be significant, whose main effects were not significant in main effects only model. However, the extension of LBL to incorporate covariates assumes haplotype-environment independence [18], and this assumption may or may not be satisfied here with age as covariate.

In the haplotype block consisting of SNPs 40 to 44 of the *ULK4* gene, the results across methods are somewhat inconsistent. LBL gives some strong association signals (with a BF >100) while haplo.score and haplo.glm results for those specific haplotypes are insignificant even though they identify some haplotypes that are not significant using LBL (see Table 2). This may be partly a result of different ways of handling missing genotype data by different software. In particular, 25 % of SNP 40′s genotypes are missing. By default, hapassoc removes any individual with more than 1 missing genotype; consequently, in this block, 60 individuals are deleted. The same deletions occur with LBL as it uses pre.hapassoc output as its input. In contrast, haplo.score and haplo.glm, by default, keep observations with some (but not all) missing genotypes by considering all possible pairs of alleles at those missing loci. There is an option in haplo.glm to exclude persons with any missing genotypes, which is not exactly the same as the hapassoc default option although close to it. We ran haplo.glm with this option for this block but found only 1 additional significant haplotype (11000; *p* value = 0.023) in this region; however, this haplotype was not one of the significant haplotypes by LBL (see Table 1). Haplo.score lacks an option to exclude persons with missing genotypes.

Here we considered a sliding window approach to explore the full gene. Alternatively, one can use a 2-stage approach by first scanning the individual SNPs and then following up with a haplotype analysis around the SNPs that are significant at a certain level in the first stage. We explored this approach by using PLINK [27] in the first stage. With an arbitrarily chosen 5 % significance level for the first stage, we found the SNPs at 41497081, 41504594, 41657184, 41841618, 41939990, 41949348, and 47956424 to be significant. The last one is on the *MAP4* gene, and the rest are on the *ULK4* gene. Comparing the results of this 2-stage approach with the results shown in Tables 1 and 3, we see that haplotypes containing all of these SNPs (not necessarily the first SNP in the block), except the SNP at 41841618, are significant by LBL. Thus, the results from the 2 types of analyses are similar. However, we note that single-SNP analysis, by itself, does not show significance in these regions, as the lowest *p* value of these 7 SNPs is 0.004; consequently, none of them achieve genome-wide significance.

We carried out all analyses on a 3.4 GHz Xeon processor under Linux operating system with 31.32 GB RAM. For sliding window analysis of *MAP4* gene, LBL takes 261 s, haplo.glm takes 53.83 s, and haplo.score takes 51.46 s. For *ULK4* gene, LBL takes 2237 s, haplo.glm takes 358.38 s, and haplo.score takes 325.39 s. Thus, gene-wide sliding window haplotype analysis is computationally feasible as a follow-up tool even with LBL.

Finally, it is noteworthy that one of the most popular collapsing method SKAT and its variants did not find significance on either gene. This suggests increased power of haplotype association methods over collapsing methods and is consistent with literature [15], but this issue needs to be evaluated fully through simulations. However, our results illustrate that haplotype association methods are useful and complement collapsing approaches not only for genome-wide association studies data but for sequencing data also, contrary to popular belief.

## Conclusions

Several haplotypes were found to be significant on the ULK4 and MAP4 genes. In particular, the haplotypes found to be significant by LBL are likely to be true positive as our results show that it maintains a low false-positive rate.
